# Combined effects of cadmium and salinity on juvenile *Takifugu obscurus*: cadmium moderates salinity tolerance; salinity decreases the toxicity of cadmium

**DOI:** 10.1038/srep30968

**Published:** 2016-08-04

**Authors:** Jun Wang, Xuexia Zhu, Xin Huang, Lei Gu, Yafen Chen, Zhou Yang

**Affiliations:** 1 Jiangsu Province Key Laboratory for Biodiversity and Biotechnology, School of Biological Sciences, Nanjing Normal University, 1 Wenyuan Road, Nanjing 210023, China; 2 State Key Laboratory of Lake and Environment, Nanjing Institute of Geography and Limnology, Chinese Academy of Sciences, 73 East Beijing Road, Nanjing 210008, China

## Abstract

Obscure puffer *Takifugu obscurus*, a species of anadromous fish, experiences several salinity changes in its lifetime. Cadmium (Cd) is a toxic heavy metal that can potentially induce oxidative stress in fish. The present study aimed to detect the combined effects of Cd (0, 5, 10, 20 and 50 mg L^−1^) and salinity (0, 15 and 30 ppt) on juvenile *T. obscurus*. Results showed the juveniles could survive well under different salinities; however, with Cd exposure, the survival rates significantly decreased at 0 and 30 ppt. At 15 ppt, tolerance to Cd increased. Cd exposure clearly induced oxidative stress, and the responses among different tissues were qualitatively similar. Salinity acted as a protective factor which could reduce the reactive oxygen species and malondialdehyde levels. In addition, salinity could enhance the antioxidant defense system, including superoxide dismutase, catalase and glutathione. Na^+^/K^+^–ATPase activity significantly decreased under Cd exposure in gill, kidney and intestine. These findings indicated that Cd could moderate the adaptability of juvenile *T. obscurus* to high salinity and low salinity played a protective role upon Cd exposure. Thus, the role of salinity should be considered when evaluating the effect of heavy metals on anadromous and estuarine fishes.

Cadmium (Cd), a non-essential heavy metal, is commonly regarded as a pollutant in aquatic habitats. With the increase in freshwater contamination by numerous natural and industrial chemical compounds, Cd has been considered an important global issue because of its persistence and capacity for bioaccumulation^1^,^2^. In polluted aquatic environments, fish are continuously exposed to ambient Cd through both water and food. Cd can cause damage to fish, including loss of appetite, reduced growth^3^, disturbance in respiratory^4^, change in hematology^5^ and disruption in whole-body or plasma ion regulation^6^ by inducing a large number of reactive oxygen species (ROS)^7^. Cd^2+^ has been reported to be unable to generate free radicals directly by itself, but can indirectly generate superoxide radicals and hydroxyl radicals^8^,^9^. Malondialdehyde (MDA) is the final product of lipid peroxidation and considered a basic compound in cellular damage by toxins, which represents direct evidence of toxicity caused by free radicals. In the long-term evolution process, aerobic biological systems have developed a mechanism to prevent peroxide damage. This mechanism includes superoxide dismutase (SOD), catalase (CAT), glutathione (GSH), and so on^10^. When fish was peroxidation damaged, those biochemical parameters were activated. Numerous studies on oxidative stress responses in fish have been conducted^11^,^12^,^13^.

Obscure puffer *Takifugu obscurus*, an ecologically and economically important fish in the Yangtze River in China, is a species of anadromous fish. *T. obscurus* migrates to freshwater rivers to reproduce during the spawning season from February to May. Newly hatched larvae remain in freshwater for several months and then move to the sea for one or two years until sexual maturity is reached. After approaching maturity, the fish return to freshwater rivers to spawn^14^,^15^. The capability of adapting to both freshwater and seawater makes *T. obscurus* a useful model species for studying osmoregulation^16^. Na^+^/K^+^–ATPase is an important membrane protein that provides the driving force for ion regulation and mediates whole-body osmoregulation among aquatic organisms. Therefore, salinity significantly affects Na^+^/K^+^–ATPase activities^17^,^18^.

The concentrations of Cd in oyster (*Crassostrea virginica*) tissue and shell were found to be 35 and 42 times higher than those of Gulf of Mexico sediment samples^19^. When macroalgae *Gracilaria lemaneiformis* were exposed to different concentrations of Cd (1, 5, 10 and 20 μg L^−1^) for 28 days, the Cd concentrations in *G. lemaneiformis* were increased 2.32, 3.42, 8.65 and 10.15 times respectively compared to those in the control (within artificial seawater)^20^. Therefore, even if the concentration of Cd in water is <5 μg L^−1^ (the Cd concentration of the second grade surface water described in the Chinese environmental quality standards for surface water: GB 3838-2002), the *T. obscurus*, which preys on shellfish and plankton with enriched Cd, is still probably damaged by Cd. Although the independent effects of Cd and salinity on *T. obscurus* have been investigated^16^,^21^, little is known about the combined effects of Cd and salinity on its survival and biochemical responses. Based on this background information, we selected *T. obscurus* juveniles as a bioindicator in the present study to investigate the potential interactions between waterborne Cd exposure and environmental salinity. We considered this study to be meaningful and proposed the following three hypotheses: (1) exposure to Cd may moderate the adaptability of juveniles to high salinity, which will be determined by observing and recording the death of juveniles daily and the changes in Na^+^/K^+^–ATPase activities; (2) sublethal waterborne Cd exposure may induce oxidative stress under different salinity levels, which will be evaluated by measuring ROS and MDA levels in various tissues of juveniles and the antioxidant enzyme activities (SOD, CAT and GSH); (3) certain salinity may reduce Cd damage, which will be evaluated by comparing survival rates, ROS levels and oxidative stress parameters among the juveniles cultured under different concentrations of Cd (0 and 5 mg L^−1^, cadmium chloride (CdCl_2_)) and salinity (0 and 15 ppt).

## Results

### Survival rates of *T. obscurus* exposed to different Cd concentrations and salinity levels

**Figure 1:**
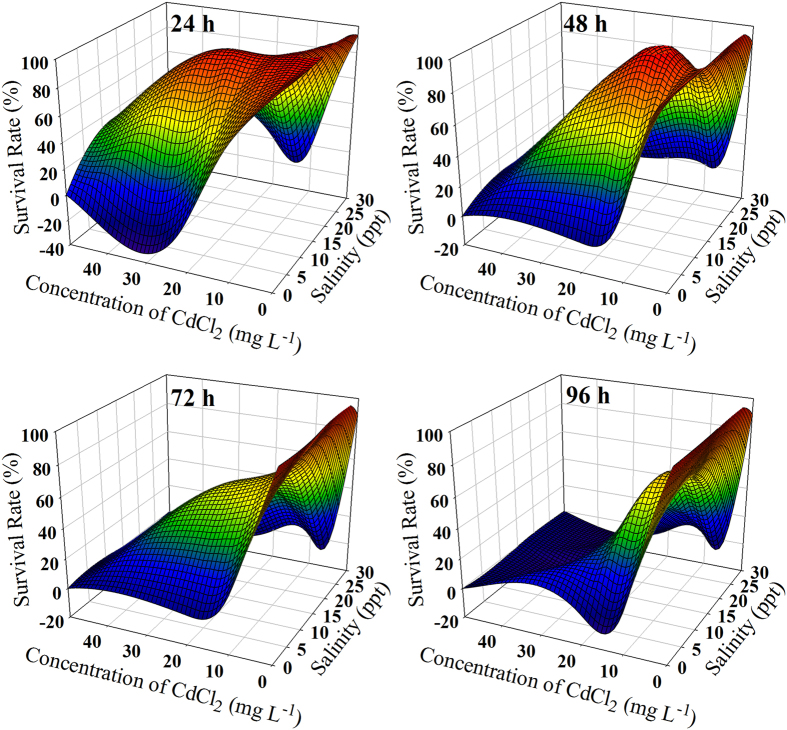
Survival rates of *T. obscurus* juveniles under different Cd concentrations and salinities at different times.

All Cd concentrations showed no significant change during the experiment (Table S1). After the juveniles were exposed to the salinity of 0 and 30 ppt for 24 h, their survival rates were decreased to 0 at the Cd concentrations of 20 and 50 mg L^−1^ (Fig. 1). However, at 15 ppt, the survival rate was above 90% under high Cd exposure (20 mg L^−1^). With increasing exposure time, the survival rates declined sharply under Cd concentrations >10 mg L^−1^ at all salinity treatments. After exposed to Cd (5 and 10 mg L^−1^) for 96 h, the survival rates were higher at salinity of 15 ppt (70% and 70%) than 0 (60% and 10%) and 30 ppt (0% and 0%). Meanwhile, the survival rates at different salinity levels without Cd exposure exceeded 90%. The Cd EC_50_ values of survival rates at 15 ppt were higher than at other salinities (Table 1) which showed that juveniles could tolerate high Cd concentrations in brackish water.

### ROS and MDA levels

**Figure 2:**
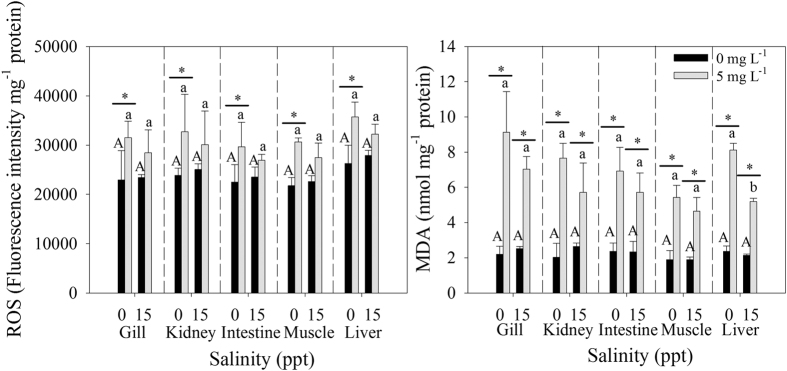
Reactive oxygen species (ROS) and malondialdehyde (MDA) in the tissues of *T. obscurus* juveniles exposed to 5 mg L^−1^ Cd at different salinities for 96 h. Capital letters indicate significant differences at different salinities without Cd in each tissue. Lowercase letters indicate significant differences at different salinities under Cd exposure in each tissue. The asterisk above black bars denotes significant difference between different Cd concentrations under the same salinity. (*P* ≤ 0.05) (n = 3).

The effects of Cd and salinity on ROS and MDA levels in each tissue were analyzed by two-way ANOVA (Table S2). Salinity itself almost had no effect on ROS and MDA levels regardless of Cd exposure (Fig. 2). Moreover, the MDA level was significantly lower at 15 ppt than at 0 ppt without Cd exposure in the liver. Upon exposure to Cd, ROS concentrations significantly increased in the gill, kidney, intestine, muscle and liver of *T. obscurus* at 0 ppt, and MDA concentrations increased significantly at 0 and 15 ppt. In general, ROS and MDA levels increased after 96 h of exposure to Cd. No significant interaction among Cd, salinity and tissue was observed (Table S3).

### Oxidative stress biomarkers

**Figure 3:**
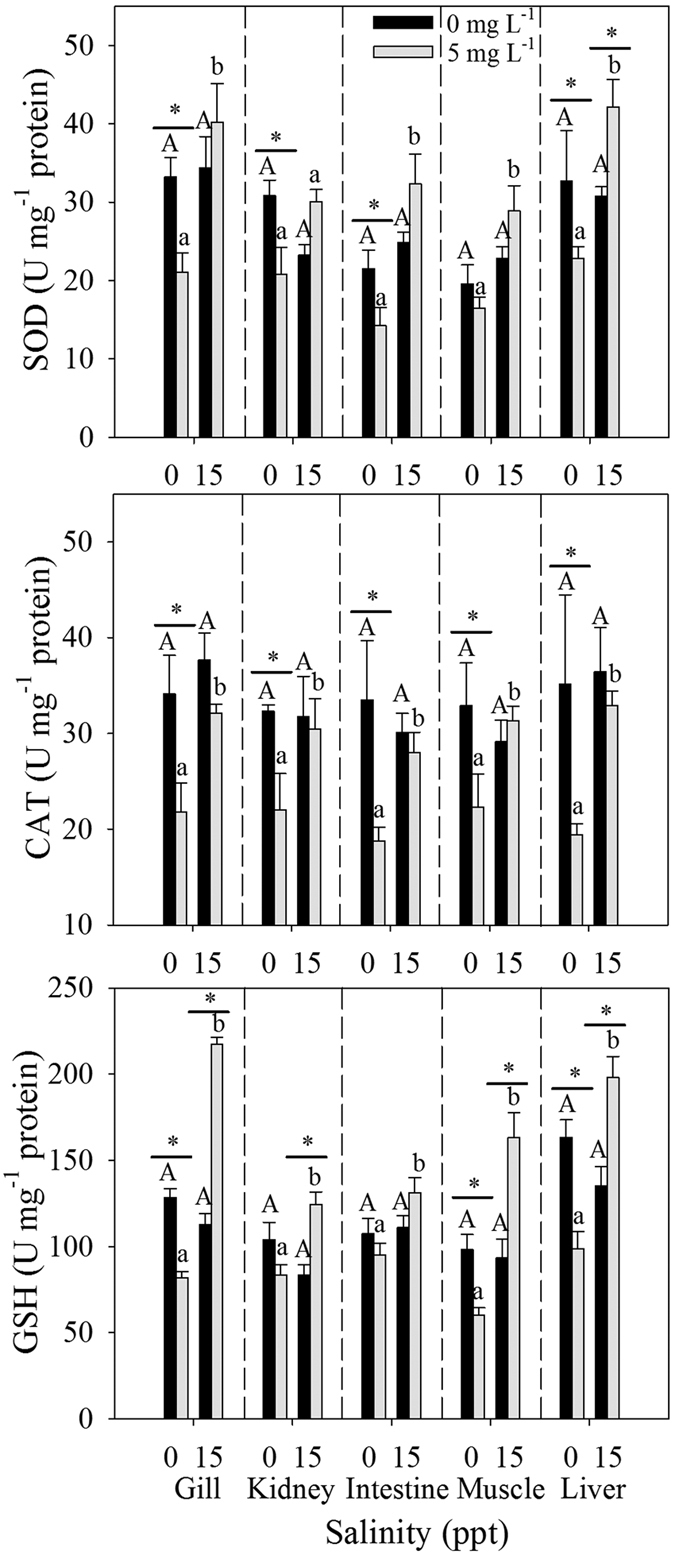
Superoxide dismutase (SOD), catalase (CAT) and glutathione (GSH) in tissues of *T. obscurus* juveniles exposed to 5 mg L^−1^ Cd under different salinities for 96 h. Capital letters indicate significant differences under different salinities without Cd in each tissue. Lowercase letters indicate significant differences at different salinities under Cd exposure in each tissue. The asterisk above black bars denotes a significant difference between different Cd concentrations under the same salinity. (*P* ≤ 0.05) (n = 3).

The response of SOD activities is illustrated in Fig. 3. Our results showed that the changes of SOD activities in all tissues were highly consistent. The SOD activities in the gill, kidney, intestine and liver significantly decreased after the juveniles were exposed to 0 ppt and Cd for 96 h. In addition, SOD activities were significantly affected by salinity under Cd exposure except in the kidney. A statistically significant interaction was found between Cd and salinity in the gill (*p* = 0.038), kidney (*p* = 0.006), intestine (*p* = 0.022) and liver (*p* < 0.001) (Table S4).

Salinity itself did not significantly affect CAT activities without Cd exposure (Fig. 3). After exposed to Cd for 96 h, CAT activities decreased significantly at 0 ppt, whereas the decrease was inhibited at 15 ppt. Subsequently, CAT activities were significantly higher at 15 ppt than at 0 ppt.

Changes in GSH levels were similar to those in SOD and CAT activities. At 0 ppt, GSH levels were inhibited significantly after the exposure to Cd in the gill, muscle and liver (Fig. 3). At 15 ppt, GSH levels were significantly higher after 96 h of exposure in all tissues except in intestine. GSH also exhibited a significant increase at 15 ppt than at 0 ppt under Cd exposure. Based on the results of two-way ANOVA, a statistically significant interaction was observed between Cd and salinity in the gill (*p* < 0.001), kidney (*p* = 0.039), muscle (*p* < 0.001) and liver (*p* < 0.001) (Table S4). A significant interaction among Cd, salinity and tissue was observed in GSH but not in SOD and CAT (Table S3).

### Na^+^/K^+^–ATPase activities

**Figure 4:**
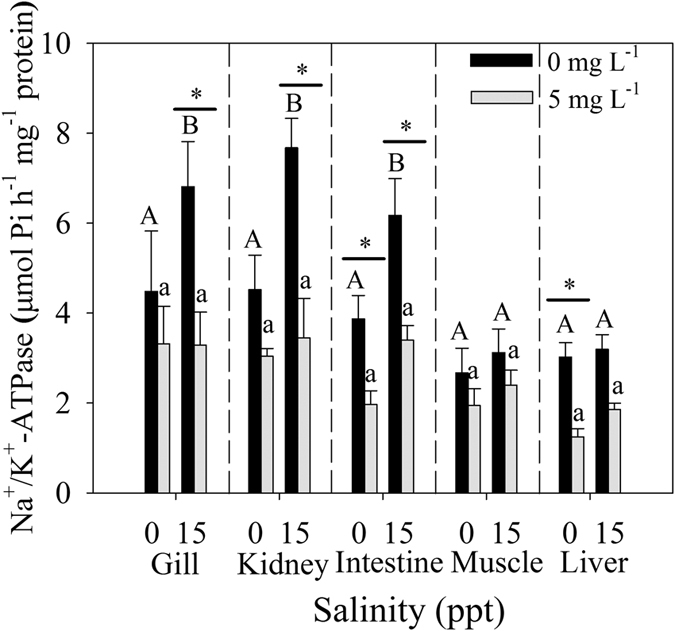
Na^+^/K^+^–ATPase in tissues of *T. obscurus* juveniles exposed to 5 mg L^−1^ Cd under different salinities for 96 h. Capital letters indicate significant differences under different salinities without Cd in each tissue. Lower case letters indicate significant differences under different salinities and Cd exposure in each tissue. The asterisk above black bars denotes a significant difference between different Cd concentrations under the same salinity. (*P* ≤ 0.05) (n = 3).

Na^+^/K^+^–ATPase activities significantly increased at 15 ppt than at 0 ppt without Cd exposure in the gill, kidney and intestine. However, after exposed to Cd for 96 h, these activities significantly decreased at 15 ppt (Fig. 4). In the intestine and liver, Na^+^/K^+^–ATPase activities exhibited a significant decrease under Cd exposure at 0 ppt. Cd significantly affected Na^+^/K^+^–ATPase activities in the gill (*p* = 0.015), kidney (*p* = 0.003), intestine (*p* = 0.002) and liver (*p* < 0.001), but only an interaction was noted between Cd and salinity in the gill (*p* = 0.039) (Table S5).

## Discussion

The present study supports the following three hypotheses: (1) Cd exposure will moderate the adaptability of *T. obscurus* to high salinity (30 ppt); (2) sublethal waterborne Cd exposure (5 mg L^−1^) would induce oxidative stress under different salinity levels; and (3) salinity acted as a protective factor in reducing Cd damage.

The contamination of Cd in aquatic systems is a consequence of industrial, agricultural, and other anthropogenic activities. Cd can accumulate in fish tissues and may become toxic for fish after tissue metal accumulation reaches a substantially in high level, depending on metal type, exposure duration, fish species, and water chemistry^22^,^23^. Our results helped evaluate the effects of Cd on *T. obscurus* survival at different salinities. *T. obscurus* can tolerate a wide salinity variation and live well in sea, river and estuary water. However, with industrial development, Cd pollution in water is considerably more serious, thereby possibly inducing combined effects with salinity on fish.

The toxicity of Cd in fish has been studied in a number of previous studies^24^,^25^. In our study, the juveniles of *T. obscurus* could survive well at different salinities. However, upon Cd exposure of juveniles, the survival rates decreased significantly at high concentrations of Cd (10, 20 and 50 mg L^−1^) at 0 and 30 ppt. As exposure time was prolonged, the survival rates continued to decline with each salinity level. After 96 h of exposure, in the lower concentration of Cd (5 mg L^−1^), the survival rate still reached 70% at 15 ppt (Fig. 1). The EC_50_ values also indicated that the juveniles could tolerate high Cd concentration at 15 ppt (Table 1). Our results confirmed that Cd was toxic to *T. obscurus*, and Cd reduced the survival rate of juveniles in freshwater and the tolerance to high salinity. However, in brackish water, tolerance to Cd increased (Table 1). Similar results also appeared on *Farfantepenaeus paulensis*^23^ showing that Cd is more toxic to *F. paulensis* at salinity of 5 ppt than at 20 and 36 ppt.

The oxidative stress in response to Cd in fish has been the focus of numerous studies^26^,^27^,^28^. The exact mechanism involved in Cd-induced oxidative stress remains unknown, but Cd is known to combine with protein molecules containing sulfhydryl, carboxyl and amino, thereby inhibiting the activities of ATPase, hydrolysis and antioxidase^29^,^30^. Another possible explanation is that Cd is a promoter of oxidative stress by inducing the formation of ROS, including the superoxide anion (O_2_^•−^), hydrogen peroxide (H_2_O_2_) and hydroxyl radical (HO^•−^)^31^. Previous studies also reported the effect of Cd exposure on the expression of antioxidant gene and metallothionein in *T. obscurus*^21^,^32^. However, the combined effect of Cd and salinity on oxidative stress in *T. obscurus* is first reported in this study.

Aerobic organisms are known to continuously produce ROS even when they simply maintain a basal level of metabolism. In this study, Salinity did not exert significant effect on ROS (Fig. 2). We also found that salinity could reduce ROS production under Cd exposure but not significantly. But ROS levels increased significantly upon exposure to Cd for 96 h at 0 ppt in all tissues. Excessive ROS lead to oxidative stress, which damage macromolecules, such as proteins, carbohydrates, nucleic acids, and lipids^33^,^34^. Therefore, the removal of excess ROS and the maintenance of a dynamic balance between the production and removal of ROS are crucial in protecting organisms from oxidative stress and maintaining normal physiological functions^35^. If the balance is disturbed, the products of this metabolism alter the normal metabolism of cells by causing oxidative stress, mainly by oxidation of proteins, DNA and lipids leading to cell death^36^. Excess ROS initiates lipid peroxidation and MDA is generated as a final product. Lipid peroxides have been considered as the basic cause of cellular damage and MDA has been used as a biomarker of oxidative stress^37^. In our study, the changes in MDA levels in different tissues were similar to those of ROS levels, and a significant increase of MDA was observed under Cd exposure (*p* < 0.05). However, the increase in MDA at 15 ppt was less than that at 0 ppt which indicated that the injury was lighter at higher salinity. The significant decreased of MDA levels were detected at 15 ppt after exposed to Cd, while it was not observed in ROS. These findings may be attributed to significantly higher SOD, CAT and GSH at 15 ppt than at 0 ppt upon Cd exposure; thus, the excess ROS were cleared.

Aquatic animals possess an evolutionary-conserved antioxidant defense system for the removal of excess ROS. This defense system is composed of antioxidative enzymes and non-enzymatic antioxidants^38^. The former includes SOD, CAT and glutathione peroxidase, and the latter includes fat-soluble vitamins and water-soluble small molecules (such as GSH and ascorbic acid). In particular, the antioxidant enzymes SOD and CAT are considered the vital first-line defenses against oxygen toxicity. When fishes are exposed to metals, SOD and CAT activities are increased^38^,^39^,^40^. In our study, SOD and CAT activities decreased significantly when the juveniles were exposed to Cd at 0 ppt (Fig. 3). This finding indicates that Cd would damage the antioxidant defense system of *T. obscurus*. The results showed that antioxidative enzymes of fishes were more active in salinity treatment than in freshwater. We also observed that SOD activity in each tissue was higher than CAT activity under Cd exposure at 15 ppt, especially in the gill and liver. It is speculated that the increase in SOD may lead to the accumulation of H_2_O_2_, which will restrain the function of CAT^41^. As the second-line of defenses against oxidative damage, GSH plays a major role in cellular metabolism and free radical scavenging^42^,^43^. In cell, GSH is also the main non-protein thiol that quenches oxyradicals via its sulfhydryl group. In addition, GSH serves as an available co-substrate for glutathione peroxidase which can catalyze the reduction of hydroperoxides into hydroxyl compounds. Our results were consistent with those described in previous studies^44^,^45^, which revealed that GSH levels in tissues decrease when organisms are exposed to Cd. In our study, GSH levels were significantly higher in salinity treatments than in freshwater under Cd exposure (Fig. 3). Individually, salinity itself did not significantly influence ROS and oxidative stress parameters, but reduced the damage caused by Cd to *T. obscurus*. A previous study speculated the mechanism of salinity protection against heavy metal zinc uptake in *Fundulus heteroclitus*^46^, which considered that salinity could alter the speciation of Zn and reduce free Zn^2+^ ion in total zinc concentration; and salinity also increased the concentration of potentially competitive cations, such as Ca^2+^ which competes with the Zn^2+^ uptake mechanism in the gills. These mechanisms may explain the changes in ROS, MDA and antioxidant enzymes in our study, but the defense of fish itself should also be taken into consideration.

The main function of transmembrane ATPase is to provide energy for ion transportation across membranes and maintain ion homeostasis in the cytoplasm. Na^+^/K^+^–ATPase is a membrane protein that facilitates the active exchange between two extracellular K^+^ ions and three intracellular Na^+^ ions as one ATP molecule is hydrolyzed. In this process, crucial K^+^ and Na^+^ concentration gradients between the cytosol and extracellular fluid are maintained^47^. Most euryhaline teleosts exhibit adaptive changes in Na^+^/K^+^–ATPase activity following salinity changes^17^,^48^. In the present study, the results were similar to the previous study that salinity significantly affects on Na^+^/K^+^–ATPase activity of juvenile *T. obscurus* in gill, kidney and intestine^18^. But when exposed to Cd for 96 h, Na^+^/K^+^–ATPase activities of the gill, kidney and intestine were significantly inhibited at 15 ppt. This finding suggested that most of the energy was used to resist against free radicals caused by Cd, and the ability of osmotic regulation was diminished. Thus, we can explain why juveniles could not tolerate high salinity (30 ppt) when exposed to low Cd concentration (5 mg L^−1^). In freshwater, Na^+^/K^+^–ATPase activity in the liver decreased significantly after 96 h of exposure to Cd, as the liver is an important organ of body that functions in detoxification. However, muscle cells cannot synthesize more Na^+^/K^+^–ATPase to maintain ion homeostasis in the cytoplasm. Overall, Na^+^/K^+^–ATPase activities of tissues were inhibited by Cd under different salinities. Certain salinity can induce high Na^+^/K^+^–ATPase activities in *T. obscurus*^18^, and previous studies confirmed that Na^+^/K^+^–ATPase activities are negatively correlated with oxidative stress^49^,^50^. Therefore, we speculated that this ATPase may also be used to reduce oxidative stress damage caused by Cd exposure. Upon exposure to high salinity, the oxidative stress damage caused by salinity and Cd was too serious for repair, thereby causing fishes death.

In summary, juvenile *T. obscurus* could tolerate high salinity (30 ppt); however this adaptability was moderated by Cd exposure. Sublethal Cd exposure significantly induced oxidative stress in *T. obscurus*. When fish were exposed to Cd for 96 h, ROS and MDA levels decreased, and the antioxidant defense system (SOD, CAT and GSH) was enhanced at 15 ppt in five tissues which suggested that salinity could reduce the damage caused by Cd to *T. obscurus* and enhanced the antioxidant defense system. Thus, within the suitable salinity and sublethal range of waterborne Cd, salinity played a protective role for juveniles under Cd exposure. Previous studies confirmed that Cd^2+^ is more toxic to *Photobacterium phosphoreum*, *Daphnia magna* and *Carassius auratus* in alkaline environments (pH = 9) than in acidic environments (pH = 5)^51^; and the toxicity of Cd to saprophytic and nitrifying bacteria decreased with increasing hardness until reached the highest concentration (32 mg L^−1^ as CaCO_3_ for nitrifying and 400 mg L^−1^ as CaCO_3_ for saprophytic bacteria^52^. These findings suggested that future toxicology studies should focus not only on the inherent toxicity of individual chemicals, but also consider possible interactions with other environmental factors. The function of salinity should be considered when evaluating the effect of heavy metals on anadromous fish, and we also consider that *T. obscurus* is a useful animal model to assess the risk of various pollutants in anadromous and estuarine fishes at different salinities.

## Materials and Methods

All experiments were approved by and carried out in accordance with the guidelines of the Institutional Animal Care and Use Committees (IACUC) of Nanjing Normal University, Nanjing, China.

### Fish collection and acclimation

The *T. obscurus* juveniles (age: two months; body length: 6.6 ± 1.2 cm; body weight: 18.5 ± 1.7 g) were obtained from a Yangzhong obscure puffer hatchery (Jiangsu, China), transferred to our laboratory and maintained in an aquarium containing 100 L of freshwater. No Cd was present in the tissues and diets of the fish. The rearing conditions in the laboratory were similar to those of the fish farm (temperature: 23 ± 1 °C; pH: 7.5 ± 0.4; light:dark = 12 h:12 h; dissolved oxygen >5 mg L^−1^; Cd concentration: <0.001 mg L^−1^). About 500 juveniles were placed in three aquariums. The commercial fish diet was supplied to satiation twice a day, without any residue left. Half of the water was renewed daily to maintain good water quality. After 7 days, healthy juveniles were selected to assess the actual effect of Cd and salinity on survival and biochemical responses in juveniles.

### Experimental design

We used a full-factorial design for Cd and salinity. The five concentrations of Cd were as follows: 0, 5, 10, 20 and 50 mg L^−1^ (CdCl_2_, Aladdin Industrial Corporation, purity 99%). The three salinities were as follows: 0, 15 and 30 ppt. Therefore, 5 × 3 combinations of treatment were established, and each treatment had three replicates. For ease of expression, the following Cd concentrations were used to indicate CdCl_2_ concentrations. Ten juveniles were placed in an aquarium filled with 30 L of test solution and continuously aerated. To acclimate to the designated salinity, we added refined industrial salt and ensured that water salinity increased 3 ppt every 6 hours until reached our pre-set salinities; the juveniles continued to adapt to 3 days in each salinity and then add different concentrations of CdCl_2_. Cd concentrations and salinity levels were monitored daily to ensure relatively constant metal and salinity exposures. Cd concentrations were analyzed using a flame atomic absorption spectrophotometer^53^, and salinity was measured by a salinometer. The temperature was 23 ± 1 °C; some other parameters were light:dark = 12 h:12 h, a dissolved oxygen concentration above 5 mg L^−1^. The number of dead juveniles was recorded and removed daily. After 96 h of exposure, one fish was randomly selected from each of these aquariums (Cd: 0 and 5 mg L^−1^; salinity: 0 and 15 ppt; triplicate). The juveniles were euthanized with a lethal dose of NaOH-neutralized MS-222. The biochemical analysis was performed on individual. Samples of gill, kidney, intestine, muscle and liver were rapidly removed by dissection, rinsed with cold physiological saline (0.6% NaCl), dried in filter paper, frozen in liquid nitrogen, and stored at −80 °C. For ROS detection, a slice of each tissue was measured immediately after rinsing and drying.

### Biochemical assays

The samples were placed in a Dounce homogenizer with 0.6% physiological saline solution and then homogenized. Subsequently, each tissue was centrifuged at 3000 rpm for 10 min at 4 °C to eliminate cellular debris and cartilage fragments. Supernatants were removed and used for biochemical parameter assays. Enzyme (SOD, CAT and Na^+^/K^+^–ATPase) activities, ROS, MDA and GSH levels in supernatants were measured using the Diagnostic Reagent Kits according to the manufacturer’s instructions (Nanjing Jian Cheng Bioengineering Institute, China). Protein concentrations were estimated using Diagnostic Reagent Kit (Coomassie protein assay dye).

### Statistical analysis

All data were expressed as treatment mean ± standard error (SE). The EC_50_ values were calculated using the SigmaPlot 11.0. The experiment was designed for analysis using repeated measures analysis of variance (two-way and three-way ANOVA) with a significance level of 0.05 to determine the effects of Cd exposure, salinity as well as their interaction on ROS, MDA, SOD, CAT, GSH, and Na^+^/K^+^–ATPase values (tested separately) in each tissue. Before ANOVA, we had conducted a homogenous test and passed it (*p* > 0.05). The post-hoc test of holm-sidak method was employed for multiple comparisons. All statistical analyses (survival rates and biochemical parameters) were carried out using SigmaPlot 11.0.

## Additional Information

**How to cite this article**: Wang, J. *et al*. Combined effects of cadmium and salinity on juvenile *Takifugu obscurus*: cadmium moderates salinity tolerance; salinity decreases the toxicity of cadmium. *Sci. Rep.* **6**, 30968; doi: 10.1038/srep30968 (2016).

## Supplementary Material

Supplementary Information

## Figures and Tables

**Table 1:** Statistical analysis of Cd EC_50_ values (mg L^−1^) of survival rates under different salinity treatments and durations.

Salinity	24 h	48 h	72 h	96 h
0 ppt	11.74	7.13	6.03	5.45
15 ppt	40.62	37.94	30.57	19.62
30 ppt	5.21	2.17	—	—

“─’’ Denotes that EC_50_ was not obtained in our study.
