# Supplementary material for: Combined effects of cadmium and salinity on juvenile Takifugu obscurus: cadmium moderates salinity tolerance; salinity decreases the toxicity of cadmium
Source: Sci Rep. 2016 Aug 4;6:30968. doi: 10.1038/srep30968 (PMC4973225; doi:10.1038/srep30968)
Supplement: Supplementary Information [file srep30968-s1.pdf]

Combined effects of cadmium and salinity on juvenile *Takifugu obscurus*: cadmium moderates salinity tolerance; salinity decreases the toxicity of cadmium

Jun Wang, Xuexia Zhu, Xin Huang, Lei Gu, Yafen Chen*, and Zhou Yang*

Table S1. The measured concentrations of Cd ion in different treatments. (n=3)

Parameter	Time (h)	Salinity 0 ppt	Salinity 15 ppt	Salinity 30 ppt
Total Cd (5 mg L^{-1}, $CdCl_2$)	24	3.04 ±0.03	3.05 ±0.03	3.04 ±0.02
	48	3.01 ±0.02	3.01 ±0.03	3.00 ±0.03
	72	2.97 ±0.03	2.98 ±0.03	2.97 ±0.02
	96	2.95 ±0.03	2.97 ±0.03	2.95 ±0.03
Total Cd (10 mg L^{-1}, $CdCl_2$)	24	6.12 ±0.01	6.12 ±0.03	6.11 ±0.02
	48	6.09 ±0.02	6.10 ±0.03	6.09 ±0.02
	72	6.05 ±0.02	6.08 ±0.02	6.07 ±0.01
	96	6.04 ±0.03	6.05 ±0.03	6.05 ±0.02
Total Cd (20 mg L^{-1}, $CdCl_2$)	24	12.23 ±0.01	12.22 ±0.03	12.22 ±0.02
	48	12.21 ±0.02	12.21 ±0.03	12.19 ±0.04
	72	12.19 ±0.02	12.18 ±0.03	12.17 ±0.02
	96	12.15 ±0.03	12.14 ±0.02	12.16 ±0.03
Total Cd (50 mg L^{-1}, $CdCl_2$)	24	30.63 ±0.02	30.64 ±0.03	30.62 ±0.04
	48	30.61 ±0.03	30.61 ±0.02	30.60 ±0.03
	72	30.58 ±0.01	30.59 ±0.02	30.57 ±0.03
	96	30.58 ±0.03	30.58 ±0.03	30.57 ±0.02

Table S2. Summary of two-way ANOVA on the interaction between Cd concentration (0, 5 mg L^{-1}) and salinity (0, 15 ppt) on ROS and MDA levels of *T. obscurus* juveniles in each tissue (n = 3).

Tissue	Parameters	Source of variation	DF	SS	MS	F	P
Gill	ROS	Cd	1	138343503	138343503	2.68	0.14
		salinity	1	4925724.6	4925724.6	0.0954	0.765
		Cd × salinity	1	9399673.4	9399673.4	0.182	0.681
	MDA	Cd	1	98.231	98.231	21.724	0.002
		salinity	1	2.369	2.369	0.524	0.49
		Cd × salinity	1	4.359	4.359	0.964	0.355
Kidney	ROS	Cd	1	145288043	145288043	1.786	0.218
		salinity	1	1469260	1469260	0.0181	0.896
		Cd × salinity	1	10690381	10690381	0.131	0.726
	MDA	Cd	1	56.998	56.998	18.246	0.003
		salinity	1	1.339	1.339	0.429	0.531
		Cd × salinity	1	4.876	4.876	1.561	0.247
Intestine	ROS	Cd	1	83934205	83934205	2.606	0.145
		salinity	1	2136318.9	2136318.9	0.0663	0.803
		Cd × salinity	1	10766983	10766983	0.334	0.579
	MDA	Cd	1	47.192	47.192	17.447	0.003
		salinity	1	1.149	1.149	0.425	0.533
		Cd × salinity	1	1.049	1.049	0.388	0.551
Muscle	ROS	Cd	1	141383547	141383547	15	0.005
		salinity	1	4314473.9	4314473.9	0.458	0.518
		Cd × salinity	1	11943948	11943948	1.267	0.293
	MDA	Cd	1	29.725	29.725	29.153	<0.001
		salinity	1	0.459	0.459	0.45	0.521
		Cd × salinity	1	0.453	0.453	0.444	0.524
Liver	ROS	Cd	1	142266415	142266415	6.901	0.03
		salinity	1	2658294.3	2658294.3	0.129	0.729
		Cd × salinity	1	19701118	19701118	0.956	0.357
	MDA	Cd	1	58.143	58.143	283.497	<0.001
		salinity	1	7.435	7.435	36.25	<0.001
		Cd × salinity	1	5.511	5.511	26.871	<0.001

Table S3. Summary of three-way ANOVA on the interaction between Cd concentration (0, 5 mg L-1), salinity (0, 15 ppt) and tissue on biochemical parameters of *T. obscurus* juveniles (n = 3).

Parameters	Source of variation	DF	SS	MS	F	P
ROS	Tissue	4	202336281.6	50584070.4	1.296	0.288
	Cd	1	645136489.2	645136489	16.523	<0.001
	Salinity	1	14794413.93	14794413.9	0.379	0.542
	Tissue × Cd	4	6079225.076	1519806.27	0.0389	0.997
	Tissue × salinity	4	709657.766	177414.442	0.00454	1
	Cd × salinity	1	61329877.87	61329877.9	1.571	0.217
	Tissue × Cd × salinity	4	1172224.831	293056.208	0.00751	1
MDA	Tissue	4	18.758	4.69	2.026	0.109
	Cd	1	279.869	279.869	120.89	<0.001
	Salinity	1	10.289	10.289	4.444	0.041
	Tissue × Cd	4	10.421	2.605	1.125	0.358
	Tissue × salinity	4	2.462	0.615	0.266	0.898
	Cd × salinity	1	13.914	13.914	6.01	0.019
	Tissue × Cd × salinity	4	2.334	0.584	0.252	0.907
CAT	Tissue	4	118.314	29.579	0.759	0.558
	Cd	1	815.553	815.553	20.918	<0.001
	Salinity	1	338.876	338.876	8.692	0.005
	Tissue × Cd	4	62.407	15.602	0.4	0.807
	Tissue × salinity	4	60.942	15.236	0.391	0.814
	Cd × salinity	1	429.485	429.485	11.016	0.002
	Tissue × Cd × salinity	4	21.619	5.405	0.139	0.967
GSH	Tissue	4	19141.078	4785.27	25.194	<0.001
	Cd	1	1183.418	1183.418	6.231	0.017
	Salinity	1	21297.198	21297.198	112.127	<0.001
	Tissue × Cd	4	2181.6	545.4	2.871	0.035
	Tissue × salinity	4	3416.914	854.228	4.497	0.004
	Cd × salinity	1	30832.177	30832.177	162.328	<0.001
	Tissue × Cd × salinity	4	8864.566	2216.141	11.668	<0.001
SOD	Tissue	4	888.65	222.163	8.552	<0.001
	Cd	1	31.94	31.94	1.229	0.274
	Salinity	1	1120.424	1120.424	43.128	<0.001
	Tissue × Cd	4	63.872	15.968	0.615	0.655
	Tissue × salinity	4	274.496	68.624	2.642	0.048
	Cd × salinity	1	1204.596	1204.596	46.368	<0.001

	Tissue ×Cd ×salinity	4	193.523	48.381	1.862	0.136
Na+/K+ ATPase	Tissue	4	56.69	14.173	12.111	<0.001
	Cd	1	57.862	57.862	49.445	<0.001
	Salinity	1	19.062	19.062	16.289	<0.001
	Tissue ×Cd	4	8.343	2.086	1.782	0.151
	Tissue ×salinity	4	5.921	1.48	1.265	0.3
	Cd ×salinity	1	4.602	4.602	3.933	0.054
	Tissue ×Cd ×salinity	4	5.924	1.481	1.266	0.299

Table S4. Summary of two-way ANOVA on the interaction between Cd concentration (0, 5 mg L^{-1}) and salinity (0, 15 ppt) on the antioxidant defense system of *T. obscurus* juveniles in each tissue (n = 3).

Tissue	Parameters	Source of variation	DF	SS	MS	F	P
Gill	SOD	Cd	1	30.399	30.399	0.773	0.405
		salinity	1	310.217	310.217	7.885	0.023
		Cd × salinity	1	241.816	241.816	6.146	0.038
	CAT	Cd	1	236.531	236.531	9.205	0.016
		salinity	1	143.372	143.372	5.579	0.046
		Cd × salinity	1	34.487	34.487	1.342	0.28
	GSH	Cd	1	2526.439	2526.439	36.253	<0.001
		salinity	1	10773.014	10773.014	154.585	<0.001
		Cd × salinity	1	17118.682	17118.682	245.641	<0.001
Kidney	SOD	Cd	1	7.988	7.988	0.529	0.488
		salinity	1	2.442	2.442	0.162	0.698
		Cd × salinity	1	211.993	211.993	14.047	0.006
	CAT	Cd	1	158.931	158.931	6.466	0.035
		salinity	1	87.714	87.714	3.569	0.096
		Cd × salinity	1	108.418	108.418	4.411	0.069
	GSH	Cd	1	36.11	36.11	0.246	0.633
		salinity	1	1711.19	1711.19	11.658	0.009
		Cd × salinity	1	886.389	886.389	6.039	0.039
Intestine	SOD	Cd	1	0.0493	0.0493	0.00244	0.962
		salinity	1	347.182	347.182	17.144	0.003
		Cd × salinity	1	163.336	163.336	8.066	0.022
	CAT	Cd	1	210.662	210.662	5.709	0.044
		salinity	1	25.817	25.817	0.7	0.427
		Cd × salinity	1	119.328	119.328	3.234	0.11
	GSH	Cd	1	43.392	43.392	0.234	0.642
		salinity	1	1186.64	1186.64	6.394	0.035
		Cd × salinity	1	804.743	804.743	4.336	0.071
Muscle	SOD	Cd	1	6.74	6.74	0.471	0.512
		salinity	1	183.932	183.932	12.852	0.007
		Cd × salinity	1	62.659	62.659	4.378	0.07
	CAT	Cd	1	103.860	103.860	4.032	0.080
		salinity	1	54.830	54.830	2.129	0.183
		Cd × salinity	1	196.332	196.332	7.622	0.025
	GSH	Cd	1	757.022	757.022	3.042	0.119
		salinity	1	7259.69	7259.69	29.17	<0.001

		Cd ×salinity	1	8683.727	8683.727	34.892	<0.001
Liver	SOD	Cd	1	83.477	83.477	2.987	0.122
		salinity	1	511.233	511.233	18.291	0.003
		Cd ×salinity	1	830.714	830.714	29.721	<0.001
	CAT	Cd	1	277.238	277.238	3.773	0.088
		salinity	1	164.418	164.418	2.238	0.173
		Cd ×salinity	1	112.457	112.457	1.531	0.251
	GSH	Cd	1	2.054	2.054	0.00688	0.936
		salinity	1	3783.578	3783.578	12.665	0.007
		Cd ×salinity	1	12203.202	12203.202	40.847	<0.001

Table S5. Summary of two-way ANOVA on the interaction between Cd concentration (0, 5 mg L^{-1}) and salinity (0, 15 ppt) on Na^+/K^+–ATPase activities of *T. obscurus* juveniles in each tissue (n = 3).

Tissue	Source of variation	DF	SS	MS	F	P
Gill	Cd	1	18.905	18.905	9.445	0.015
	salinity	1	11.822	11.822	5.907	0.041
	Cd × salinity	1	12.156	12.156	6.074	0.039
Kidney	Cd	1	24.436	24.436	17.972	0.003
	salinity	1	9.526	9.526	7.006	0.029
	Cd × salinity	1	5.634	5.634	4.144	0.076
Intestine	Cd	1	16.441	16.441	19.408	0.002
	salinity	1	10.413	10.413	12.292	0.008
	Cd × salinity	1	0.575	0.575	0.678	0.434
Muscle	Cd	1	1.573	1.573	3.268	0.108
	salinity	1	0.612	0.612	1.272	0.292
	Cd × salinity	1	4.87E-08	4.87E-08	1.01E-07	1
Liver	Cd	1	7.278	7.278	50.702	<0.001
	salinity	1	0.452	0.452	3.146	0.114
	Cd × salinity	1	0.143	0.143	0.996	0.347
